# Intracerebroventricular Administration of ^192^IgG-Saporin Alters Expression of Microglia-Associated Genes in the Dorsal But Not Ventral Hippocampus

**DOI:** 10.3389/fnmol.2017.00429

**Published:** 2018-01-17

**Authors:** Yulia V. Dobryakova, Artem Kasianov, Maria I. Zaichenko, Mikhail Y. Stepanichev, Ekaterina A. Chesnokova, Petr M. Kolosov, Vladimir A. Markevich, Alexey P. Bolshakov

**Affiliations:** ^1^Institute of Higher Nervous Activity and Neurophysiology, Russian Academy of Sciences, Moscow, Russia; ^2^Vavilov Institute of General Genetics, Russian Academy of Sciences, Moscow, Russia; ^3^Pirogov Russian National Research Medical University, Moscow, Russia

**Keywords:** ^192^IgG-saporin, RNA-seq, dorsal hippocampus, ventral hippocampus, microglia, rat, behavior

## Abstract

One of important aspects of development of Alzheimer’s disease is degeneration of septal cholinergic neurons that innervate the hippocampus. We took advantage of widely used model of cholinergic deficit in the hippocampus, intracerebroventricular administration of ^192^IgG-saporin (Ig-saporin), to analyze the postponed consequences of cholinergic deficit in different parts of the hippocampus. We studied effects of the immunotoxin on the behavior of rats and gene expression in the dorsal and ventral hippocampus using RNA-seq approach. We found that under normal conditions dorsal and ventral parts of the hippocampus differ in the expression of 1129 protein-coding genes and 49 non-coding RNAs (ncRNAs) and do not differ in the expression of 10 microRNAs, which were detected in both parts of the hippocampus. Ig-saporin-induced degeneration of cholinergic septal neurons did not affect rat behavior in open field, T-maze, and passive avoidance task but impaired memory retention in Morris water maze. To analyze ^192^Ig-saporin-induced changes in the gene expression, we formed the following groups of genes: genes expressed exclusively in certain cell types (neurons, astrocytes, microglia, oligodendrocytes, and vascular cells) and, among universally expressed genes, a group of genes that encode ribosome-forming proteins. For all groups of genes, the alterations in the gene expression produced by the immunotoxin were stronger in the dorsal as compared to the ventral hippocampus. We found that, among groups of universally expressed genes, Ig-saporin increased the expression of ribosome-forming proteins in both dorsal and ventral hippocampus. Ig-saporin also strongly upregulated expression of microglia-specific genes only in the dorsal hippocampus. A subset of affected microglial genes comprised genes associated with inflammation, however, did not include genes related to acute inflammation such as interleukins-1b, -6, -15, and -18 as well as TNF. The expression of other cell-specific genes (genes specific for neurons, astrocytes, oligodendrocytes, and vascular cells) was unaffected. The data obtained suggest that disturbance of memory-associated behavior after administration of Ig-saporin is associated with upregulation of microglia-associated genes in the dorsal but not ventral hippocampus.

## Introduction

Alzheimer’s disease (AD) is a devastating disease which strongly reduces cognitive abilities. Hundreds of studies on the mechanisms of development of this disease revealed some critical processes that occur during AD; however, there is no clear understanding which factors are the most critical in pathology development. Currently, it is known that AD is associated with accumulation of insoluble beta-amyloid plaques in the brain; however, accumulation of these plaques does not always correlate with development of AD (Herrup, 2015). It is also known that AD is associated with accumulation of neurofibrillary tangles of hyperphosphorylated tau (Grundke-Iqbal et al., 1986; Takashima, 2013). It was also shown that AD causes strong alterations in the expression of genes (Tan et al., 2010; Berchtold et al., 2014; Wang et al., 2016) but the key factors that induce these changes are not clear.

Another important event that occurs in AD is degeneration of cholinergic neurons in the basal nuclei including septal neurons that form inputs to the hippocampus (Francis et al., 1999). Since neurons of the septum and diagonal band of Broca (DBB) are the only source of cholinergic innervation in the hippocampus, degeneration of these cells results in a strong decrease in the functioning of all acetylcholine-dependent systems. It was shown that cholinergic septal input to the hippocampus regulates excitability and oscillatory activity of hippocampal network, and its degeneration impairs hippocampal network activity [reviewed in (Teles-Grilo Ruivo and Mellor, 2013)]. However, network activity is determined not only by architecture and functioning of synapses but also by intrinsic properties of all cells that form and support network, including glial and endothelial cells. So far, there is no evidence that, in addition to its effect on hippocampal network activity, long-term cholinergic deficit may also alter the expression of genes critical for normal functioning of hippocampal cells. It was shown that activity of metabotropic acetylcholine receptors may regulate expression of some genes (Von Der Kammer et al., 1999; Albrecht, 2000); hence, it is possible to hypothesize that disruption of cholinergic innervation of the hippocampus may alter expression of genes that are critical for functioning of hippocampal cells. To analyze consequences of cholinergic deficit in the hippocampus at the mRNA level, we used a well-described model of induction of cholinergic deficit in the hippocampus – intracerebroventricular (i.c.v.) injection of immunotoxin ^192^IgG-saporin (Ig-saporin) which causes selective degeneration of cholinergic neurons in the septum and DBB (Heckers et al., 1994).

^192^IgG-saporin is a conjugate of antibody to NGF receptor, which is predominantly expressed on cholinergic septal neurons, and a ribosomal toxin saporin, which after penetration into a cell causes irreversible damage of ribosomes and cell death. Ig-saporin-induced degeneration of cholinergic neurons is associated with development of cognitive deficit in different behavioral tasks (Waite et al., 1995; Calza et al., 2003; Janisiewicz et al., 2004; Jeong et al., 2014; Knox and Keller, 2016).

The majority of previous studies on the effects of Ig-saporin was predominantly focused on the analysis of behavioral or electrophysiological consequences of degeneration of cholinergic neurons (Kuczewski et al., 2005; Kanju et al., 2012). Only a few studies analyzed effects of Ig-saporin on the expression of genes in the neocortex and hippocampus (Paban et al., 2010, 2011a). However, the analysis performed in these studies did not take into account specific features of different parts of the hippocampus. Here, we studied changes caused by Ig-saporin in the gene expression in the dorsal and ventral hippocampus of rats using RNA-seq approach. We analyzed ventral and dorsal parts of the hippocampus separately since these parts of hippocampus strongly differ in the expression of a large number of genes (Fanselow and Dong, 2010; Cembrowski et al., 2016) and involved in different types of learning (Moser et al., 1995; Fanselow and Dong, 2010). For instance, dorsal part mediates spatial navigation and memory formation while the ventral part is involved in anxiety and affective responses. Furthermore, these parts of the hippocampus receive cholinergic innervation from different structures (Fanselow and Dong, 2010). Using currently available databases on cell-specific expression of genes in the brain (Zhang et al., 2014; Zeisel et al., 2015), we analyzed cell-specific changes produced by i.c.v. injection of Ig-saporin in the ventral and dorsal parts of the hippocampus.

## Materials and Methods

The experiments were performed with adult male Sprague-Dawley rats (250–350 g) received from Research Center of Biomedical Technology RAMS, nursery “Pushchino.” A total of 16 rats were involved in the study (*n* = 8/group). Animals were housed under standard vivarium conditions at 21 ± 1°C with a 12 h light/dark cycle, food and water were provided *ad libitum*. All experiments were performed in accordance with the ethical principles stated in the EU Directive 2010/63/EU for animal experiments and were approved by the Ethical Committee of the Institute of Higher Nervous Activity and Neurophysiology of the Russian Academy of Sciences.

### Stereotaxic Surgery and Drug Administration

Rats were anesthetized with chloral hydrate (400 mg/kg, i.p.) and mounted in a Kopf stereotaxic frame. Since there is a great variability in the Ig-saporin doses that induce substantial loss of septal cholinergic neurons, we performed pilot experiments where Ig-saporin was injected at doses 0.5, 1, 2, and 4 μg/site. In our hands, only a dose of 4 μg/site induced a strong loss of cholinergic neuron in the septum. Rats received bilateral i.c.v. infusions of saporin (4 μg/site). Hamilton syringe (Hamilton Company, United States) was lowered into the ventricle (0.8 mm posterior, 1.5 lateral to bregma). Drug (4 μl/site) was infused by a microinfusion pump (Stoelting Co., United States) at a rate of 0.2 μl/min. Rats were allowed to recover for 21 days after the surgery. The sequence of behavioral testing was as follows:

(i) days 21–31, Morris water maze; (ii) day 32, open-field test; (iii) days 33–37, T-maze; (iv) days 38–39, passive avoidance (PA) training. The samples of brain tissue were collected on day 41.

### Morris Water Maze Testing (Days 21–31)

The water maze consisted of a circular pool (diameter 1 m; height 60 cm) filled with water at a temperature of 22–24°C up to a height 30 cm. It was virtually divided in four equal quadrants identified as north (N), east (E), south (S), and west (W). A platform (10 × 10 cm) was placed in the center of one of the quadrants and was 1.5–2 cm under the water surface. For each trial, a rat was placed at a starting point in the pool facing the wall. After each trial, rats were placed under a heating lamp to prevent hypothermia.

Testing procedure involved two daily trials for 60 s. For each trial, the rat was given a maximum of 60 s to reach the hidden platform, which was located in the center of the SW quadrant. The pseudorandom order of the two possible starting points was changed from day to day. When the rat climbed on the platform, it was allowed to stay there for 10 s. When a rat failed to find the platform within 60 s, it was guided to it and allowed to stay there for 10 s. Inter-trial interval was about 30 min for each rat. On day 31, the platform was removed from the pool and all the rats were given a 60-s probe trial. This delayed probe trial is used to evaluate the strength and precision of an established spatial memory by the amount of the time spent in the target quadrant. The following parameters were recorded: the latency to reach the platform, total distance moved, the swimming velocity, and tracks. Behavior was recorded automatically using Ethovision Software (Noldus, Wageningen, Netherlands).

### Open-Field Test (Day 32)

Exploratory and emotional behavior was studied in a round open field. This arena (diameter of 1 m) was surrounded with a wall (height 50 cm). The arena was divided in three equal concentric zones (central, mid, and peripheral). Each rat was placed in the center of the arena and the rat’s behavior was recorded automatically for 5 min under red light using Ethovision software (Noldus, Wageningen, Netherlands). The following behavioral variables were quantified: total distance moved (centimeter), velocity (centimeter/seconds), movement frequency, total movement duration (seconds), vertical activity (rearings), grooming, and number of entries to the center of the arena.

### T-Maze Training (Days 33–37)

The T-maze apparatus consisted of a central arm (20 × 10 cm) and two arms (30 × 10 cm). On the first day of experiment rats were placed into the central arm and allowed to move freely for 20 min for habituation. After the habituation period, the rats were food-deprived. The next 4 days the rats were released from the starting position and the time taken to reach the goal compartment (containing a reward of several food pellets) was measured, as well as the number of wrong entries. Testing procedure involved five daily trials of 60 s. For each trial, the rat was given a maximum of 60 s to reach the goal compartment. Inter-trial interval was about 30 min for each rat. To avoid odor cues, the maze was properly cleaned with 70% ethanol between trials.

### Passive Avoidance (PA) Training (Days 38–39)

The test apparatus (OpenScience, Russia) consisted of a plastic box divided into two equal compartments (30 cm × 30 cm × 30 cm): one was white-colored and brightly illuminated and the other one was black-colored and dark. The two compartments were not separated by door.

During the first trial, rat was placed into the light compartment and allowed to move freely between the two parts of the chamber for 5 min (habituation trial). After the habituation trial, the rat was placed into the same chamber (acquisition trial), behavioral conditions were similar to the habituation trial but entry into the dark compartment was paired with a 10-s electric shock (0.5 mA) provided through the metal grid covering the floor of the test camera.

After the shock, rat was immediately removed from the apparatus. In 24 h, during the retention trial, no foot shock was given and the step-through latency was recorded as a measure of retention.

### Tissue Preparation for Analysis

Rats were anesthetized with chloral hydrate (400 mg/kg, i.p.) 1.5 month after the injection. They were then submitted to intracardiac perfusion with ice-cold 0.9% NaCl. The brains were removed and the frontal part of the brain, which contains septum, was dissected and post-fixed in 4% paraformaldehyde (Sigma–Aldrich, United States) solution in 0.1 M PBS (Biolot, Russia) for at least 2 days. The rest of the brain was cut along the midline and, in the left half, we isolated dorsal and ventral parts of the hippocampus (the hippocampus was cut into three equal parts, the middle part was discarded, the other parts were considered as dorsal and ventral, respectively) and took them for further analysis. Hippocampal samples were immediately transferred to vials and frozen in liquid nitrogen.

### Immunohistochemistry and Cell Count

The full extent of the septum was sectioned at 50-μm-thick coronal brain sections using a vibrating blade microtome (Leica VT1200 S, Germany). Six sections from the septum area were selected according to a random sampling scheme and stained for ChAT using a 2-day protocol. In brief, slices were incubated in 0.3% triton X-100 (SERVA, Germany) in 0.01 M PBS (PBS-T) three times for 5 min at room temperature, then for 1 h in blocking solution consisting of 5% normal goat serum (Sigma–Aldrich, United States) in PBS-T and later in blocking solution with primary antibodies (rabbit anti-Choactase 1:500, Santa Cruz Biotechnology, United States) at 4°C overnight. The next day, sections were washed in PBS-T and incubated with secondary antibodies (1:800, goat anti-rabbit IgG-biotin, Sigma–Aldrich, United States) in blocking solution at room temperature for 1 h. After additional washing in PBS, the sections were incubated with avidin–biotin–HRP complex (ABC Elite kit, Vector Labs, United States) for 1 h, and 3,3′-diaminobenzidine (SIGMA-Fast Kit, Sigma–Aldrich, United States) was used as a chromogen for development of staining. All images were acquired with a Leica DM6000B microscope (Leica, Germany) or Keyence BZ-9000 (Keyence, Japan). Imaging parameters were set to avoid signal saturations.

Delineation of brain structures was performed in images according to Paxinos and Watson atlas (Paxinos and Watson, 1998). The first section was randomly chosen at +1.2 mm from bregma and then, six sections located 150 μm from each other were chosen for analysis. All stained cell within the medial septum and DBB profiles were counted. Only cells located within one focal plane were counted in order to prevent overestimation. The mean number of cells per section was counted and was considered as a representative value for one animal.

### RNA-Seq

Total RNA was isolated from the hippocampal samples using ExtractRNA Reagent (Evrogen, Russia) following manufacturer’s protocol. Before preparation of libraries, total RNA in the samples was analyzed using an Agilent 2100 Bioanalyzer to confirm purity of RNA isolation and the absence of RNA degradation. In all samples, RIN > 8. For RNA-seq analysis, we took four rats from the control and Ig-saporin-treated groups of animals.

For depletion of ribosomal RNA, we used a NEBNext^®^ rRNA Depletion Kit (Human/Mouse/Rat) in accordance with manufacturer’s protocol. Then we prepared cDNA libraries using an Ion Total RNA-Seq Kit v2 for Whole Transcriptome Libraries (Ion Torrent, Life Technologies) following manufacturer’s protocols. After mRNA fragmentation, mRNA was purified using magnetic beads (Magnetic Bead Cleanup Module, Ion Torrent, Life Technologies). Concentration of fragmented RNA was measured using a NanoDrop 2000.

For reverse transcription, RNA was ligated with probes complementary to the primer used for reverse transcription (Ion RT Primer v2) and, then, reverse transcribed with a SuperScript^®^ III Enzyme Mix. The obtained cDNA was purified using a Magnetic Bead Cleanup Module. To amplify purified cDNA, we performed PCR using a Platinum^®^ PCR SuperMix High Fidelity Mix with forward barcode-primers from a Ion Xpress^TM^ RNA-Seq Barcode 01-16 Kit and reverse primer Ion Xpress^TM^ RNA 3′-Barcode Primer. The number of PCR cycles was maximum shown in manufacturer’ protocol. Amplified cDNA was purified using Magnetic Bead Cleanup Module. The quality of each prepared cDNA library was evaluated using NanoDrop 2000, Qubit^®^ 2.0 Fluorometer (with Qubit^®^ dsDNA HS Assay Kit), and Agilent 2100 Bioanalyzer (with High Sensitivity DNA Chip and Agilent High Sensitivity DNA Kit). In our samples, amount of short cDNA fragments with length of 25–160 bp did not exceed 10%.

During the next step, we performed clonal amplification using an Ion PI^TM^ Template OT2 200 Kit v3 and an Ion OneTouch^TM^ 2 System (Life Technologies) in accordance with manufacturer’s recommendations. After amplification, the samples were centrifuged, and precipitates were resuspended in bidistilled water and kept at +4°C for 14–18 h.

Sequencing was performed using an Ion PI^TM^ Sequencing 200 Kit v3 and an Ion PI^TM^ Chip v2 on an Ion Proton^TM^ sequencer. One chip contained four libraries with different barcodes.

Raw reads were mapped on transcriptome of rat genome Rnor 6.0 version by using BWA aligner (Li et al., 2009). Raw read counts were evaluated by SAMtools software (Li et al., 2009). Set of differentially expressed (DE) genes were estimated by DeSeq2 (Love et al., 2014).

List of genes that are selectively expressed in different cells types was created on the basis of data of single cell RNA-seq (Zhang et al., 2014; Zeisel et al., 2015). Genes that were not included in cell-specific sets (i.e., were expressed in more than one subpopulation of brain cells) were marked as universal. Three subgroups of genes were selected among the universal genes according to cholinergic synapse and ribosome reference pathways from KEGG database. DE genes list was analyzed for subsets of genes specific to tissues and specific to selected pathways. In this type of analysis, DE genes were selected if adjusted *P*-value of DeSeq2 test <0.05.

Raw read data were published to SRA and can be accessed by using range of accession numbers SRR5750530–SRR5750542.

### Statistical Analysis

All data are presented as mean ± SEM. Across groups of behavioral data, statistical significance between means was determined using a factorial repeated measures analysis of variance (ANOVA) followed by Fisher’s LSD *post hoc* test to reveal group differences on separate time intervals. The differences in behavioral activities of the rats in the round open fields, T-maze, and PA test were tested with Student’s *t*-test. *P*-values < 0.05 were considered as statistically significant.

## Results

### Effects of Intracerebroventricular Injection of Ig-Saporin on the Behavior of Rats and Expression of Choline Acetyltransferase in Cells of the Medial Septum

**FIGURE 1:**
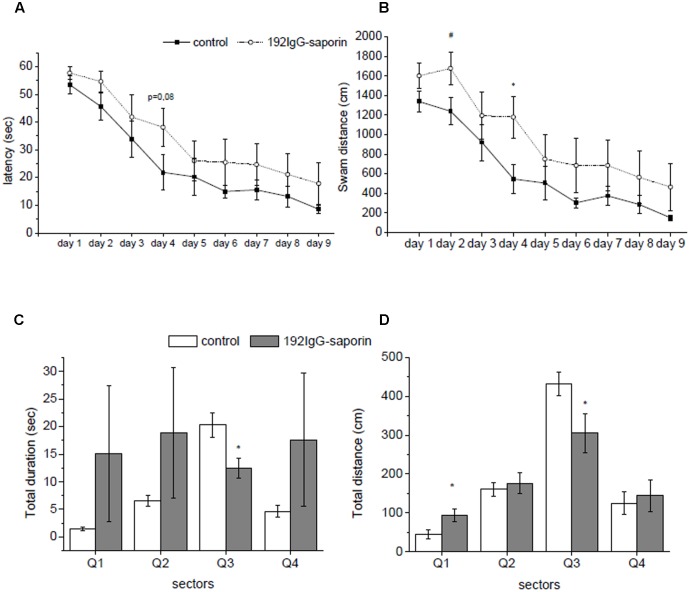
The effects of intraventricular injection of ^192^IgG-saporin (Ig-saporin) on spatial memory in Morris water maze task. Ig-saporin injected rats (*n* = 8) had longer latencies to reach the platform (seconds) **(A)** and higher distance swam (centimeter) **(B)** compared to control (*n* = 8) during the 9 days when the animals learned to find platform. On day 10 of testing, during probe trial saporin-treated rats (*n* = 8) spent significantly less time in a quadrant, where the platform was during training **(C)**, and swam shorter distance in it compared to the control (*n* = 8) **(D)**. Each point represents the mean ± SEM, ^∗^*P* < 0.05.

In order to estimate the effects of Ig-saporin on brain functioning, the control and toxin-treated animals were trained to perform spatial navigation task in the Morris water maze. Averaged latency to find a hidden platform within the training period was analyzed. Although the animals exhibited a significant decrease in the latency to find a platform from Day 1 to Day 9 of training (*F*_8,112_ = 20.35, *P* < 0.001 according to ANOVA with repeated measures), the effect of lesion was not significant (group effect *F*_1,14_ = 3.5, *P* = 0.08) (**Figure 1A**). However, the animals from the immunotoxin-treated group exhibited a strong trend to swim longer distance to reach the platform as compared to the control group (group effect *F*_1,14_ = 4.03, *P* < 0.06), and this distance decreased during the training period in both groups studied (*F*_8,112_ = 20.5, *P* < 0.001) (**Figure 1B**). Furthermore, the immunotoxin-treated rats tended to swim more rapidly as compared to the control animals (group effect *F*_1,14_ = 1.9, *P* = 0.19) probably indicating their hyperactivity under the stressful conditions of maze training. Interestingly, we did not reveal any significant interactions between group × day factors for any of indices of learning in the water maze.

A closer look to behavioral patterns that were exhibited by rats during training revealed two time periods. During first 5 days rats learned new skill, and during the rest of training period they just reproduced known skill, and analysis of data related simultaneously to both periods may obscure effect of the immunotoxin. To evaluate whether Ig-saporin influenced learning processes, we analyzed the data for the first 5 days separately. We found that from day 1 to day 5 of training the animals exhibited a significant decrease in the latency to find a platform (*F*_4,56_ = 14.7, *P* < 0.001 according to ANOVA with repeated measures) and the effect Ig-saporin was insignificant (group effect *F*_1,14_ = 3.1, *P* = 0.1). However, in contrast to the above data on the period of 10 days, analysis of behavior during first 5 days showed that the Ig-saporin-treated animals swam longer distance to reach the platform as compared to the control group (group effect *F*_1,14_ = 5.44, *P* < 0.05). Furthermore, the immunotoxin-treated rats swam more rapidly as compared to the control animals (group effect *F*_1,14_ = 4.3, *P* = 0.057) and this may explain why the latencies were similar in these two groups.

On day 10, a probe trial without platform was performed in order to test whether the animals established long-term memory on its previous location. We found that immunotoxin-treated rats spent significantly shorter period of time in the maze quadrant, where the platform was located during training (*P* < 0.01, **Figure 1C**), and swam shorter distance within the target quadrant (*P* < 0.05, **Figure 1D**) as compared to the control animals. These data show that in spite of the minor differences in the indices of learning observed during training the rats of the Ig-saporin group could not form memory on platform location in the pool.

We also evaluated locomotor activity of rats in the “open-field” test. We found that locomotor and exploratory activity did not differ between the experimental groups. Next, we studied learning abilities of animals in a T-maze where rats learned to find positive reinforcement. The only significant difference between lesioned and control rats was a decrease in the time spent by lesioned animals to leave the start corridor (*P* < 0.05, Mann–Whitney *U*-test, **Supplementary Figure S1**), which was observed during the first training day. Except this difference, analysis of animal behavior in this task did not reveal any significant differences between lesioned and control rats.

The last behavioral test that we used in our study was PA training. We found that Ig-saporin induced a trend to a decrease in the step-through latency as compared to the control animals (109.4 ± 45.3 s in Ig-saporin-treated rats vs. 160.9 ± 52.8 s in the control *P* < 0.5) (**Supplementary Figure S2**).

We found that administration of the immunotoxin caused a significant decrease in the number of ChAT-positive neurons in the septum and DBB (**Supplementary Figure S3**). Taken together, our data on behavioral and immunohistochemical experiments suggest that Ig-saporin induced mild memory impairments, which were probably associated with loss of cholinergic neurons of the medial septum/DBB complex.

### Transcriptomic Analysis of Effects of Ig-Saporin on the Gene Expression in the Ventral and Dorsal Hippocampus

We performed RNA-seq followed by differential expression analysis to detect genes whose expression was altered in the dorsal and ventral parts of the hippocampus 1.5 month after i.c.v. administration of Ig-saporin and a day after the last behavioral testing. First, we compared the expression of genes between the dorsal and ventral hippocampus under normal conditions; second, we analyzed Ig-saporin-induced changes in the expression of genes in the dorsal and ventral hippocampus.

First, we analyzed differential expression of genes in the dorsal and ventral hippocampus to validate our RNA-seq approach. We found that 1178 genes are DE in the dorsal and ventral parts: 1129 protein-coding genes and 49 ncRNAs. Our results on the protein-coding genes completely correspond to the previous data on differences in the gene expression between the dorsal and ventral parts of the hippocampus (Fanselow and Dong, 2010) and recent RNAseq analysis performed in mice (Cembrowski et al., 2016). In addition, we extended the previous data by adding a set of 49 ncRNAs whose expression differ between the ventral and dorsal parts of the hippocampus. These data are summarized in Supplementary Excel Spreadsheet (**Supplementary Table S1**).

**FIGURE 2:**
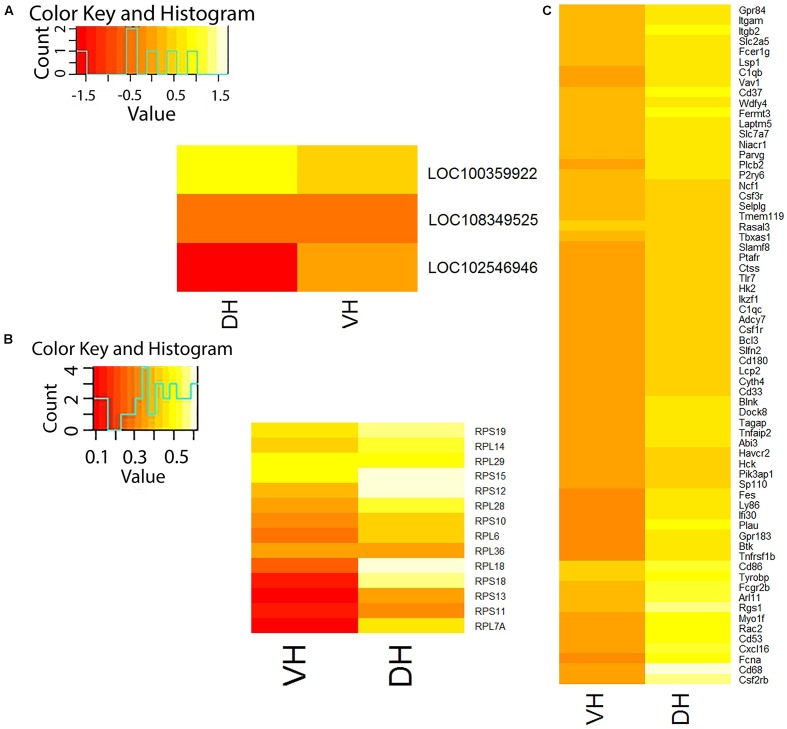
Differentially expressed genes in the dorsal (DH) and ventral (VH) hippocampus after treatment with Ig-saporin. **(A)** Heatmap shows differential expression of non-coding RNAs in VH and DH. **(B)** Heatmap shows differential expression of ribosome-forming genes in VH and DH. **(C)** Heatmap shows differential expression of microglia-specific genes in VH and DH. It may be seen that DH is stronger affected by Ig-saporin than VH.

According to our RNA-seq data, only 10 of 438 currently annotated miRNAs are expressed in the hippocampus of adult rats (Mir155hg, Mir3084d, Mir770, Mir3577, Mir1949, Mir3597-2, Mir568, Mir1843b, Mir3064, and Mir664-2). We found that the expression of these miRNAs does not depend on the part of hippocampus and Ig-saporin also did not affect their expression in both parts of the hippocampus. We also analyzed changes produced by Ig-saporin in the expression of ncRNAs and found that the immunotoxin altered the expression of putative ncRNAs LOC108349525 and LOC102546946 in the dorsal hippocampus and LOC100359922 in the ventral hippocampus (**Figure 2A**). However, since functional role of these ncRNAs is unclear, currently, it is hard to interpret these results.

Next, we analyzed changes that occurred in the hippocampus 1.5 month after the induction of degeneration of cholinergic septal neurons. This time point was chosen to avoid possible effects associated with the process of degeneration of cholinergic axons in the hippocampus which may occur at early stages after injection of Ig-saporin. In general, RNA-seq analysis revealed very small changes [Log2(Fold change) < 1.5] in the expression of all genes in different parts of the hippocampus, which is similar to previously reported magnitude of changes after Ig-saporin (Paban et al., 2010). To analyze how changes in the gene expression are related to some cell subpopulations and cellular processes, we subdivided genes into several groups. According to Zhang et al. (2014) and Zeisel et al. (2015), there are some genes that are highly selectively expressed in specific cell types in the brain. For our further analysis, we took groups of genes that are selectively expressed in neurons (500), astrocytes (115), oligodendrocytes (45), microglia (249), and vascular cells [pericytes, vascular smooth muscles, and endothelium (224)]. Other genes were considered as universal genes because they are expressed in more than one cell subpopulation. Among universal genes, we considered only genes that are related to acetylcholine transmission (nicotinic and muscarinic acetylcholine receptors, acetylcholine and choline transporters) and ribosomal proteins (48).

#### Universal Genes

Detection of DE genes in our RNA-seq data revealed only two universal genes in the dorsal hippocampus (Mt3, RGD1359290) and one gene (Rfng) in the ventral hippocampus whose expression was significantly changed after Ig-saporin. Mt3 is a universal protein that is actively involved in Zn/Cu-exchange in cells and regulation of autophagy (Lee and Koh, 2010) and has neuroprotective role (Santos et al., 2012). The gene RGD1359290 encodes a protein that putatively has relation to ribosome functioning due to some homology to ribosomal proteins in its sequence.

#### Ribosome-Forming Proteins

Next, we studied Ig-saporin-induced alterations in the expression of ribosomal proteins. We found that the expression of 14 and 6 genes (out of 48), which encode ribosome-forming proteins, increased in the dorsal hippocampus and the ventral hippocampus, respectively (**Figure 2B**).

#### Acetylcholine-Related Genes

We found that, among both muscarinic and nicotinic acetylcholine receptors, Chrna4 was the only gene whose expression in the hippocampus was affected by Ig-saporin-induced degeneration of cholinergic fibers. Its expression significantly decreased in the dorsal but not ventral hippocampus. These data suggest that the expression of almost all acetylcholine receptors does not depend on the incoming acetylcholine activity but rather an intrinsic property of hippocampal cells. The fact that hippocampal cells preserve sensitivity to acetylcholine after immunotoxin-induced lesioning is supported by finding that non-selective muscarinic agonist oxotremorine can reverse impairments caused by Ig-saporin (Van Kampen and Eckman, 2010).

#### Cell-Specific Genes

Analysis of differential expression of neuron- and oligodendrocyte-specific genes as well as genes that are specifically expressed in the vascular cells did not reveal any changes neither in dorsal nor in ventral part of the hippocampus.

Treatment with Ig-saporin resulted in an increase in the expression of two of astrocyte-specific genes in the hippocampus: complement protein C4b in the dorsal hippocampus and mitochondrial thiosulfate sulfurtransferase Tst in the ventral hippocampus.

#### Microglia-Specific Genes

Analysis of differential expression of microglia-specific genes showed that Ig-saporin altered the expression of a large number (66) of these genes only in the dorsal hippocampus whereas the ventral hippocampus remained practically unaffected (expression of only one gene increased) (**Figure 2C**). Note that the level of expression of all responded genes increased and there were no genes whose expression decreased after Ig-saporin in both parts of the hippocampus. Out of 66 genes, 7 genes are related, according to PANTHER database (Mi et al., 2017), to formation of inflammatory response: Itgb2, Plcb2, Bcl3, Rgs1, Ptafr, Rac2, and Vav1. Note, however, we did not detect any signs of acute inflammation, like increased expression of IL1b, TNF, and IL6.

## Discussion

In this study, we analyzed consequences of Ig-saporin-induced degeneration of cholinergic inputs to the hippocampus at the level of gene expression. According to our behavioral data, analysis of gene expression was performed in rats with moderate impairments of learning and memory. Alterations in animal behavior observed in the Morris water maze suggest that toxin treatment predominantly affected structures involved in spatial navigation. It has been shown that different parts of the hippocampus are involved in different types of learning. The dorsal hippocampus is critical for spatial learning (Fanselow and Dong, 2010), and lesion of this, but not ventral, part of the hippocampus by injection of ibotenic acid causes severe deficits in spatial learning in a water maze (Moser et al., 1995). Taken together, these data suggest that Ig-saporin might have impaired functioning of the dorsal hippocampus which led to disturbances in spatial navigation in Morris water maze.

The major aim of the present study was to elucidate the genes whose expression is dependent on the presence of cholinergic innervation and cell subpopulations that are strongly affected by cholinergic degeneration; the latter can give us probable targets for correction of AD-like disorders. We expected that the immunotoxin-induced degeneration will induce changes in the gene expression that, at least partly, coincide in the dorsal and ventral hippocampus; this will give us basis for making suggestions on the acetylcholine-dependent regulation of gene expression. Instead of this, we found that changes produced by i.c.v. injection of Ig-saporin are different in the dorsal and ventral parts of the hippocampus. According to analysis at the level of cell-specific genes, the strongest effect of Ig-saporin was observed in the dorsal part whereas the ventral hippocampus was practically unaffected. Furthermore, this conclusion corresponds to our above suggestion that degeneration of cholinergic input to the hippocampus mostly affects the dorsal hippocampus, which leads to the development of specific learning deficits. According to our morphological data, Ig-saporin induced degeneration of both medial septal neurons, which innervate the dorsal hippocampus, and neurons in the DBB, which send projections predominantly to the ventral part of the hippocampus. If the effects of Ig-saporin were associated only with degeneration of cholinergic fibers it will be hard to expect large difference between responses in the ventral and dorsal parts of the hippocampus. These data suggest that effects induced by Ig-saporin are related not only to degeneration of cholinergic inputs but also to some additional effects of Ig-saporin. One of possible explanations of asymmetric effect of the immunotoxin is that the dorsal hippocampus is located very close to the cerebral ventricles where we injected the immunotoxin and, as a consequence, Ig-saporin diffused predominantly into this part of the hippocampus and induced stronger effects in it.

Our data suggest that the ventral hippocampus is practically insensitive to Ig-saporin and a comparison of gene expression in the control and Ig-saporin-treated animals revealed only minor changes in this structure. This means that long-term behavioral training that was performed in our study caused similar changes in the expression in the ventral hippocampus in the control and experimental rats and a comparison of these changes revealed only subtle differences. This is not the case for the dorsal hippocampus where we found alterations in the gene expression between lesioned and control rats. Obviously, the changes in the expression of genes in the DH are related to some alterations caused by the immunotoxin. Of course, we cannot exclude possibility that behavioral procedures caused different changes in the expression of genes in DH in the control and experimental animals but, obviously, these differences occurred as a result of application of the immunotoxin.

The effect of Ig-saporin on the expression of genes in the dorsal hippocampus was quite different from the Ig-saporin effects described by Paban et al. (2010) in the entire hippocampus. In general, the sets of genes affected by Ig-saporin in our study and previous studies (Paban et al., 2010, 2011b) do not coincide. First, the mentioned studies did not include the complete list of altered genes (only the genes that were altered by learning are presented) and it is hard to be absolutely sure that the altered genes that we detected in our study are absent from their list. Second, the results obtained in Paban et al. (2011b) do not correspond to the results in Paban et al. (2010), which the authors related to limitations of different technical approaches used in their studies. Third, we administered Ig-saporin into the ventricles whereas in the mentioned studies, the immunotoxin was injected into the medial septum and nucleus basalis magnocellularis. Our data suggest that intracerebroventricularly administered Ig-saporin diffuses not only in the medial septum, where it induced degeneration of cholinergic neurons, but also, probably, in the dorsal hippocampus. Fourth, we analyzed separately effects produced by Ig-saporin in the dorsal and ventral hippocampus, which was not performed in the mentioned studies. Fifth, we analyzed subset of cells, where the changes were caused by Ig-saporin, on the basis of currently available databases (Zhang et al., 2014; Zeisel et al., 2015) of cell-specific expression of genes, which was not performed in the discussed studies.

We found that, among universal genes, Ig-saporin increased expression of a group of universal genes that encode ribosome-forming proteins. Their expression was altered in both dorsal and ventral hippocampus with largest effect observed in the dorsal part. This effect of Ig-saporin may be associated, on the one hand, with degeneration of cholinergic fibers and, on the other hand, with side effect of the toxin. Saporin *per se* inactivates ribosomes and, therefore, an increase in the expression of ribosomal proteins may be just a compensation of this action of saporin in the cells that endocytosed the immunotoxin but did not die. The specificity of Ig-saporin action is determined by antibody conjugated with saporin, however, it is unclear whether this complex immunotoxin may be endocytosed by cells that do not express Ngfr, for example, by activated microglia, which functions as macrophages. At least, we cannot exclude this possibility. On the other hand, cholinergic signaling may be one of factors that modulate processes of ribosome synthesis in hippocampal cells, however, so far there is no data on acetylcholine-dependent regulation of ribosome synthesis.

Our major unexpected findings were that lesion of cholinergic neurons practically did not influence expression of genes that are selectively expressed in all types of hippocampal cells except microglia. Previous data suggest that Ig-saporin induces a rapid activation of microglia in the septum (Rossner et al., 1995; Seeger et al., 1997). It also was shown that minocycline, which inhibits microglial activity, has a protective effect against Ig-saporin-induced degeneration of cholinergic septal neurons (Hunter et al., 2004). The authors showed that damaging effect of Ig-saporin was associated with activation of microglia in the septal area. In our study, we did not perform morphological analysis in the hippocampus; however, our transcriptomic data suggest that Ig-saporin induced postponed elevation of a large number of microglia-specific genes in the dorsal hippocampus which may result from microglia activation and/or proliferation. Note that we studied effects of Ig-saporin 1.5 months after its injection when all processes of microglial activation (if they occurred in the hippocampus after the injection) should already cease. We did not find any signs of acute microglia activation such as overexpression of pro-inflammatory interleukins (Il1B, Il6, Il15, and Il18) or TNF-alpha. This means that an increase in the expression of microglia-related genes may be a result of microglia activation that still resided in the dorsal hippocampus after acute microglia activation induced by Ig-saporin injection. This residual upregulation of microglial genes may be not only a consequence of inflammation that occurred during degeneration of cholinergic fibers but also result from deficit of acetylcholine, which is known to suppress inflammatory processes (Carnevale et al., 2007).

Finally, we found that i.c.v. injection of Ig-saporin leads to a mild cognitive deficit and is associated with very weak changes in the expression of genes. Our transcriptomic data suggest that the deficit was associated with upregulation of a number of microglia-specific genes, including inflammation-related genes, suggesting that altered functioning of microglia is among factors that at cellular level influenced cognitive abilities of animals. It was shown that microglia may influence functioning of synapses by dendritic pruning or engulfing synapse elements, especially under pathological conditions (Hong et al., 2016b). It was shown that mild cognitive impairment in a model of AD is associated with elevated expression of proteins related to complement system by microglial cells which results in strong synaptic loss (Hong et al., 2016a). Probably, under our conditions, Ig-saporin-induced activation of microglia disturbed normal functioning of hippocampal synapses which led to development of mild cognitive deficit.

Thus, our results of RNA-seq analysis suggest that i.c.v. injection of Ig-saporin strongly upregulates expression of microglia-related genes and impairs behavior associated with functioning of the dorsal hippocampus. We believe that alterations in the expression of microglia-specific genes in the part of the hippocampus, which is responsible for spatial navigation, and impairments in spatial navigation after treatment with Ig-saporin are closely related phenomena. It appears that development of mild cognitive deficit may result not from alterations in functioning of neurons but from upregulation of microglia functioning alone. In fact, we observed a situation when microglia overexpress a number of inflammation-related genes without signs of acute inflammation and these changes are practically “ignored” by other cell types. This means that induction of cholinergic deficit in the hippocampus finally leads to the development of postponed processes in microglia and activation of specific cascades, which differ from cascades associated with acute inflammation. This is important for understanding of mechanisms of the development of pathological processes, such as AD.

## Author Contributions

YD contributed in intracerebroventricular injections, design and performance of behavioral experiments, analysis of behavioral data, preparation of brain slices, immunohistochemistry, and writing manuscript. AK contributed to analysis of transcriptomic data. MZ contributed in intracerebroventricular injections, design, and performance of behavioral experiments. MS contributed to analysis of behavioral experiments, analysis of immunohistochemical data, and writing manuscript. EC contributed in preparation of samples for RNA-seq, RNAseq experiments. PK contributed in design and performance of RNAseq experiments. VM contributed in general design of study and writing manuscript. AB contributed in general design of study, analysis of transcriptomic data, and writing manuscript.

## Conflict of Interest Statement

The authors declare that the research was conducted in the absence of any commercial or financial relationships that could be construed as a potential conflict of interest.

## Supplementary Material

**FIGURE S1 |** Effect on IgG-saporin on learning in T-maze. Graph shows the time spent in the central arm of the maze. Rats that received IgG-saporin (*n* = 8) spent smaller time in the central arm as compared to the saline-treated rats (*n* = 8) only during the first trial. Bars represent mean ± SEM. ^∗^*P* < 0.05.

**FIGURE S2 |** Effect of IgG-saporin on passive avoidance learning. Trained IgG-saporin-treated rats (*n* = 8) tended to decrease latency to enter the dark chamber compared with trained saline rats (*n* = 8). Bars represent the mean ± SEM. ^∗^*P* < 0.05.

**FIGURE S3 |** Immunocytochemical staining of cholineacetyltransferase (ChAT) in the control and Ig-saporin-treated animals **(A)**. The results of counting of ChAT-positive neurons in the septum and diagonal band of Broca **(B)**. Differences between the control and experimental animals are significant in both structures (Student’s *t*-test, *P* < 0.05).

**TABLE S1 |** Differential expression of genes between the dorsal and ventral hippocampus of control rats. The data are presented as ratio DH/VH; “+” means higher expression in the dorsal hippocampus.
