# Supplementary figures and images for: Intracerebroventricular Administration of 192IgG-Saporin Alters Expression of Microglia-Associated Genes in the Dorsal But Not Ventral Hippocampus
Source: Front Mol Neurosci. 2018 Jan 17;10:429. doi: 10.3389/fnmol.2017.00429 (PMC5776139; doi:10.3389/fnmol.2017.00429)

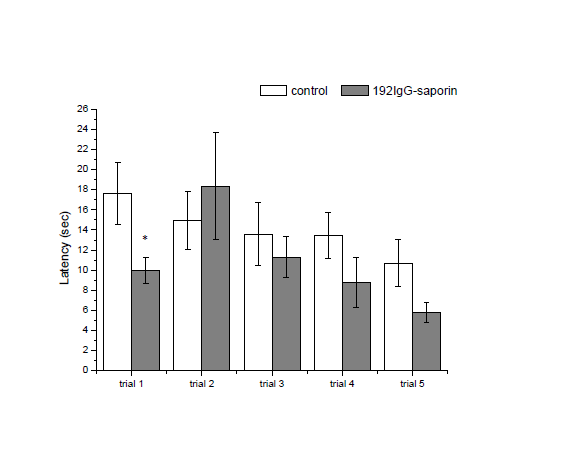

Supplement: Supplementary file 1 [file Image_1.TIF]

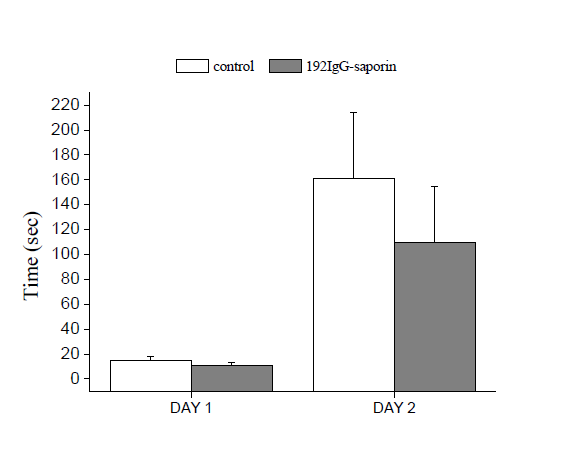

Supplement: Supplementary file 2 [file Image_2.TIF]

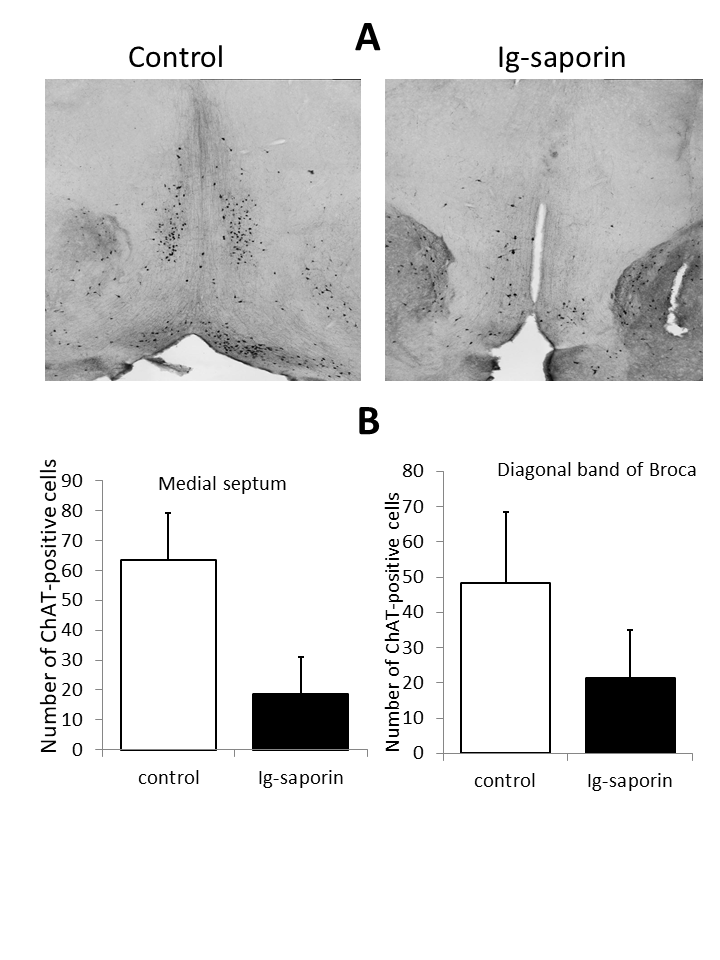

Supplement: Supplementary file 3 [file Image_3.TIF]
